# A systematic evaluation of copy number alterations detection methods on real SNP array and deep sequencing data

**DOI:** 10.1186/s12859-019-3266-7

**Published:** 2019-12-24

**Authors:** Fei Luo

**Affiliations:** School of Computer Science, Wuhan University, Wuhan, China

**Keywords:** Tumor, Copy number alterations, SNP array, Deep sequencing

## Abstract

**Background:**

The Copy Number Alterations (CNAs) are discovered to be tightly associated with cancers, so accurately detecting them is one of the most important tasks in the cancer genomics. A series of CNAs detection methods have been proposed and new ones are still being developed. Due to the complexity of CNAs in cancers, no CNAs detection method has been accepted as the gold standard caller. Several evaluation works have made attempts to reveal typical CNAs detection methods’ performance. Limited by the scale of evaluation data, these different comparison works don’t reach a consensus and the researchers are still confused on how to choose one proper CNAs caller for their analysis. Therefore, it needs a more comprehensive evaluation of typical CNAs detection methods’ performance.

**Results:**

In this work, we use a large-scale real dataset from CAGEKID consortium to evaluate total 12 typical CNAs detection methods. These methods are most widely used in cancer researches and always used as benchmark for the newly proposed CNAs detection methods. This large-scale dataset comprises of SNP array data on 94 samples and the whole genome sequencing data on 10 samples. Evaluations are comprehensively implemented in current scenarios of CNAs detection, which include that detect CNAs on SNP array data, on sequencing data with tumor and normal matched samples and on sequencing data with single tumor sample. Three SNP based methods are firstly ranked. Subsequently, the best SNP based method’s results are used as benchmark to compare six matched samples based methods and three single tumor sample based methods in terms of the preprocessing, recall rate, Jaccard index and segmentation characteristics.

**Conclusions:**

Our survey thoroughly reveals 12 typical methods’ superiority and inferiority. We explain why methods show specific characteristics from a methodological standpoint. Finally, we present the guiding principle for choosing one proper CNAs detection method under specific conditions. Some unsolved problems and expectations are also addressed for upcoming CNAs detection methods.

## Introduction

Copy Number Variations (abbreviate it to CNVs) is one kind of genomic structural variation defined as a gain or loss region in size over 1 kb. Recently, CNVs have been found to be linked to complex traits in humans and have tight relationship [1] with the transcriptome [2] and gene expression [3]. Through being present in the functional genomic regions, CNVs could affect gene dosage, gene disruption and gene fusion. Different from other molecules’ association with cancers [4–6], copy number change involves in the initiation and development of cancers in a way that copy numbers are different in an individual’s germline DNA and in the DNA of a clonal sub-population of cells. Such copy number change is specially called as somatic CNAs (copy number alterations) [7, 8]. The difference between CNAs and CNVs is that copy number alterations are changes in copy number that have arisen in somatic tissue and copy number variations originate from changes in copy number in germline cells. Many oncogenes and tumor suppressors are associated with the CNAs [9–11]. In non-small-cell lung cancer, heavy smoking patients have significantly copy number gains in 8q and 12q [12]. Copy number gain of gene EGFR is associated with the HER2-positive breast cancer [13]. Besides disease-driven genes, CNAs also harbor tumor related miRNAs [14, 15]. Two international research organizations TCGA (the cancer genome atlas) [16] and ICGC (international cancer genome consortium) [17] are dedicating to collecting and deeply interrogating the variants of typical cancers’ genomics. In the both of them, the CNAs is a hotspot to understand the cancer etiology.

Over decades, the SNP array (single nucleotide polymorphism array) and the aCGH array (array comparative genomic hybridization array) have been widely applied to detect CNAs [18, 19]. Due to probes’ low-resolution, these two micro-array platforms are suitable for CNAs research in a population. Deep sequencing offers an alternative way to discover CNAs in any size [20]. Nowadays, deep sequencing has four possible strategies to translate the mapping configuration of short reads to the CNAs findings. The first one makes use of the read depth information. The number of reads mapped to certain position of the genome is proportional to the DNA copy number. Any copy number deviation from the normal state will be reflected as increment (gain) or decrement (loss) of the read count. The second one is based on the paired-end reads. One DNA fragment is sequenced from both ends, which are called as paired end reads. If no variant occurs in the region marked by this pair of reads, their mapping positions should satisfy with the restrictions of the distance, strand and direction. The third one is based on the read split. Through examining whether one read is split into two discontinuous parts, the breakpoints of a variant could be identified. Due to the limitation of read length, paired-end and read split are only suitable for discovering the Indel variants. The final strategy is the de novo assembly. In theory, the de novo assembly could detect any structural variant, but the inherent computational insufficiency is the obstacle for its widely application.

Notable, the CNVs and CNAs are two completely different biological concepts. The CNVs and CNAs detection procedures for SNP based methods are usually different. However, sequencing based methods seldom consider genotype information, partly because of no enough sequencing depth and accuracy to detect aneuploidy and LOH (Loss of Heterozygosity). When only using the read depth information, the CNVs and CNAs detection procedures for sequencing based method are quite similar.

A series of CNAs detection methods have been proposed and several attempts have been made to compare the existing methods. Mosén [21] compared six CNAs detection methods (ASCAT, GAP, GenoCNA, GPHMM, MixHMM and OncoSNP) based on SNP array data. Magi [22] discussed six read counts based CNAs detection methods (RDXplorer, ReadDepth, CNAseg, CNV-seq, JointSLM and CNVnator). Duan [23] did the similar work on the methods CNV-seq, FREEC, SegSeq, ReadDepth, CNVnator and RDXplorer. Alkodsi [24] compared 12 methods (BICseq, HMMcopy, CNAnorm, SegSeq, COPS, CNAseg and rSW-seq for WGS data, ExomeCNV, VarScan2, ADTEx for WES data, and ControlFreeC for WGS WES). However, there are some limitations in these comparison works. Firstly, most evaluations are implemented on the synthetic datasets or small-scale real datasets. The reliability of conclusions greatly relies on how well the hypothesis to generate the synthetic dataset approximates to the real scenarios. Even though on real data, the comparisons just focus on several chromosomes of one sample, rather than at samples level. Secondly, using inappropriate way to compare those methods that need different data. For example, CNVseq needs normal sample as reference to call somatic CNAs, whereas ReadDepth, CNVnator, and RDXplorer only depend on tumor sample to call both germline and somatic variants by the GC-content correction. The matched samples and the GC-content correction for only tumor sample couldn’t place on equal footing, because using tumor-normal pair could not only eliminate the GC-content bias but also the mappability and systematic errors. Magi and Duan both generate synthetic normal samples for those methods that need matched samples. Finally, some methods phased out. For instant, the alignable coordinates file of SegSeq [25] only updates to hg18. CNAseg [26] has some errors in source code.

Although new CNAs detection methods are being proposed, they still use above methods to prove their own performance [27, 28]. Furthermore, the practical genomic structural variation analysis projects are incline to use typical CNAs methods to ensure the conclusion’s comparability and authority [29, 30]. Therefore, in this work we exclude the outdated methods and focus on those typical methods. All methods will be evaluated on the renal clear cell carcinoma dataset, which contain the tumor and matched normal samples detected not only by the SNP array but also by the deep sequencing. It guarantees the evaluation’s fairness and objectivity.

## Methods

### Typical methods selected to compare

According to mosén’s comparison conclusion, GAP and GPHMM work best and are respectively recommended to the professional and general users. The OncoSNP could simultaneously use the tumor and normal samples information. We take the GAP, GPHMM and OncoSNP as the representatives of the SNP based methods. The ReadDepth, CNVnator, RDXplorer and CNVseq are mentioned in both works [22, 23]. These four methods are also included in our work. JointSLM is discarded for it’s special for CNAs detection on a group of samples. Referring to the list given by Xi [31] and Alkodsi [24], another five methods are added into our work.

The Table 1 illustrates all 12 methods’ key features, which are BICseq [32], CNVnorm [33], FREEC [34], CNV_seq [35], rSWseq [36], Varscan [37], CNVnator [38], ReadDepth [39], RDXplorer [40], GPHMM [41], GAP [42], OncoSNP [43]. First six methods are sequencing based methods that need tumor-normal pair samples. CNVnator, ReadDepth and RDXplorer are also sequencing based methods that only need tumor sample. Last three ones are methods working on SNP array.

**Table 1:** The key features of 12 methods

Method	Window size	Normalization	Segmentation	Contamination	Ploidy
BICseq	Manual	Ratio centralization (built-in)	Bayesian information criterion	No	No
CNVnorm	Manual	GC, smoothing, Ratio centralization (built-in)	Circular binary segmentation	Yes	Yes
FREEC	CV and Poisson distribution	GC, mappability, Ratio centralization (built-in)	LASSO and dynamic programming	Yes	No
CNV_seq	Gaussian Ratio and Geary-Hinkley transformation	Ratio centralization (manual)	Consecutive Overlapping windows	No	No
rSWseq	No need	Ratio centralization (manual)	Smith-Waterman	No	No
Varscan	Fixed length broke by the gap and significant change	Ratio centralization (manual)	Circular binary segmentation	No	No
*CNVnator*	Manual	GC	Mean shift algorithm	No	No
*ReadDepth*	Negative binomial	GC, mappability	Circular binary segmentation	No	No
*RDXplorer*	Manual	GC	Event wise testing	No	No
*GPHMM*	–	–	HMM	Yes	Yes^a^
*GAP*	–	–	Circular binary segmentation	Yes	Yes
*OncoSNP*	–	–	HMM	Yes	Yes^a^

^a^They don’t directly give the ploidy estimation in the output file, but through baseline shift and exact copy number results the ploidy is indirectly known

### Dataset

Renal Cell Carcinoma (RCC) data from CAGEKID (the CAncer GEnomic of the KIDney) consortium [44] are used to compare the total 12 methods. CAGEKID is a part of International Cancer Genome Consortium (ICGC). Renal cell carcinoma accounts for approximate 3% of the adult malignancies in the worldwide [45]. RCC has four subtypes, including clear cell, papillary, chromophobe and collecting duct renal cell carcinoma. The clear cell carcinoma takes up about 80% of RCC. RCC is one of the tumor types for which there are currently no biological markers in the routine clinical use and there are few treatment options due to its inherent resistance to chemotherapy and radiotherapy. So far, the renal cell carcinoma of clear-cell type (ccRCC) has been reported significant 3p deletion and chromosome 5 and 7 amplification and some sporadic CNAs on other chromosomes [46].

**Fig. 1:**
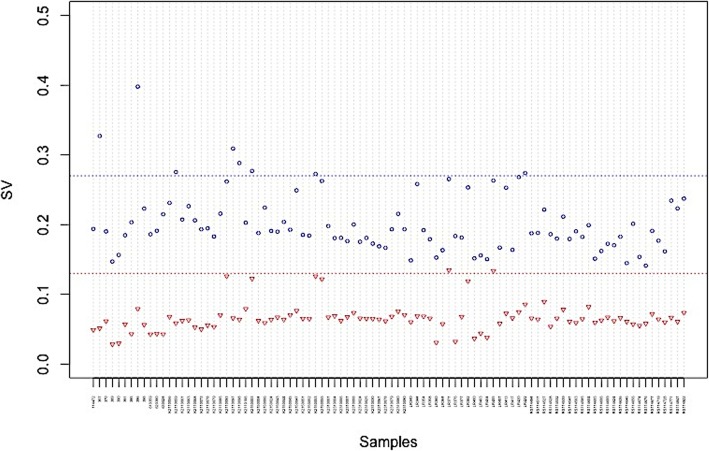
The standard deviations of LRR and BAF for 94 RCC patients’ samples. The circle symbol is the LRR standard deviation and the triangle symbol is the BAF standard deviation

We collect SNP array data of 94 RCC patients’ samples. They are genotyped by Illumina 660 W quad BeadChip, which has more than 657,000 genetic probes. To evaluate a dataset whether has a good quality, two standard deviation thresholds 0.27 and 0.13 for LRR (Log R Ratio) and BAF(B Allele Frequency) are recommend [47]. Shown in the Fig. 1, most of samples meet these thresholds. The standard deviation of BAF is smaller than that of LRR, indicating that the CNAs’ genotype states are more difficult to distinguish than the copy number states. The standard deviations of 94 samples’ LRR and BAF vary widely enough to ensure comprehensive comparison. The patients’ samples in CAGEKID project are sequenced by Illumina HiSeq 2000. The mean length of the read is 100 bp. All 10 samples’ reads are aligned to NCBI 37 reference genome by the BWA.

The 12 methods have different output descriptions for the copy number. The SNP based methods, CNVnorm, FREEC, RDXplorer and ReadDepth output exact copy number value, while the other methods output copy number gain or loss. In order to unify their output format, all copy number results are transformed into the copy number gain or loss according to the following formula. Because of the aneuploidy [48] and the SNP array experiment protocol that the genome DNA amounts hybridized to the array are the same for all samples, the ploidy value rather than the absolute copy number 2 should be used as the threshold to separate the copy number gain and loss for the tumor samples.

$$ \left\{\begin{array}{c}\frac{copy\ number\ value}{ploidy\ value}>1, gain\\ {}\frac{copy\ number\ value}{ploidy\ value}<1, loss\end{array}\right. $$

### Rank SNP based methods

For the tumor genomes, the chromosomal aneuploidy, stromal contamination, and intratumoral heterogeneity are three major obstacles to accurately detect the CNAs. Depending on the genotype information, the SNP based methods are more reliable in estimating large-scale CNAs than the sequencing based methods. Thus GAP, GPHMM and OncoSNP are firstly compared, and then the best caller will be used as the gold standard to evaluate the sequencing based methods.

In order to evaluate the SNP based methods’ performance, we propose two criteria to rank GAP, GPHMM and OncoSNP.

1. correct estimation for the baseline shift,
2. correct estimation for large length CNAs.

The aneuploidy is a frequently appearing phenomenon in tumor genomes. It always makes the LRR baseline of SNP array result shift away from zero. Criterion one emphasizes that the accuracy of LRR baseline shift estimation is the prerequisite for correctly assigning copy number gain or loss. The number of probes in a SNP chip is insufficient to discover short length CNAs, but large length CNAs take up most fraction of total CNAs length in a sample. Meanwhile, large size CNAs could be validated by visualization. Therefore, the second criterion can reflect CNAs calling accuracy. According to these two criteria, three grades are generated.

$$ \left\{\begin{array}{c} if\ satisfying\ both\ 1\  and\ 2, grade\ 1\\ {} if\ satisfying\ 1\  but\  not\ 2, grade\ 2\\ {} if\ not\ satisfying\ 1, grade\ 3\end{array}\right. $$

**Fig. 2:**
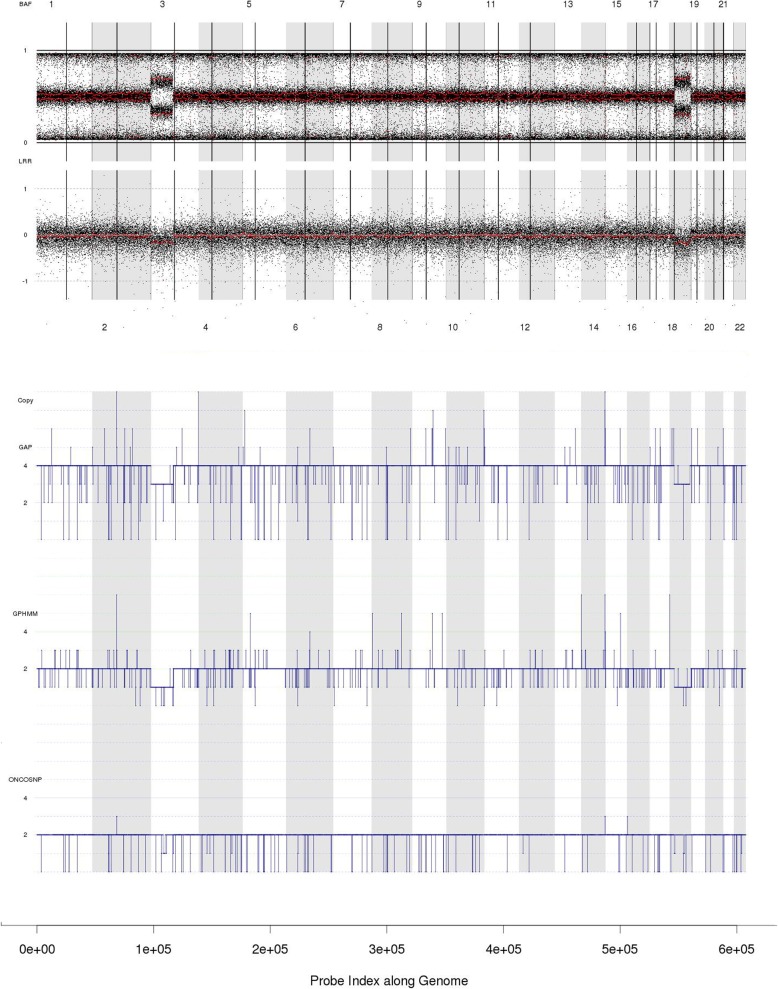
An example demonstrates the process of ranking three methods’ results. GAP wrongly judges the ploidy, so it’s graded as 3. OncoSNP loses the 3p and chromosome 18 deletions but has correct ploidy estimation. Thus it’s graded as 2. GPHMM has correct ploidy estimation and no chromosome-scale CNAs error, so it gets score 1

When visually examining how three SNP based methods satisfy with the criteria, raw LRR, BAF and the CNAs outputs from three methods are depicted in one figure, as shown in Fig. 2. All results are validated by the expert’s visual examination. The best SNP based method’s results will be used as benchmark.

### Evaluate sequencing based methods

**Fig. 3:**
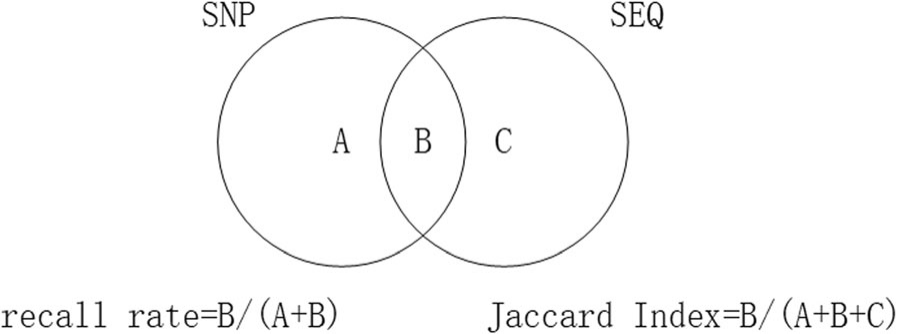
The recall rate and the Jaccard index used to measure the consistency of the SNP based benchmark and the sequencing based method

Since results from the SNP based methods are sufficient to cover large events and most fraction of total CNAs in one sample, the consistency with the best SNP based result could reflect one sequencing based method’s performance. We use the recall rate and the Jaccard index to evaluate the degree of consistency between the SNP based benchmark and the sequencing based method. The Jaccard index is a statistic for comparing the similarity and diversity of two sets. As shown in the Fig. 3, recall rate could measure how much part of the SNP based benchmark is covered by the sequencing based method. It reflects the true positive rate of the sequencing based method. In theory, the sequencing based method could discover more short CNAs benefitting from its higher resolution, but the size of total short CNAs only takes up a small part. If the Jaccard index is too low, it means that the sequencing based method has a high false positive rate.

## Results

### Evaluation of SNP based methods

#### Grades

When affected by the tumor samples’ aneuploidy, the LRR baseline representing average copy number would shift away from zero. Precise LRR baseline shift estimation is the basis of copy number alteration judgment [49].

**Fig. 4:**
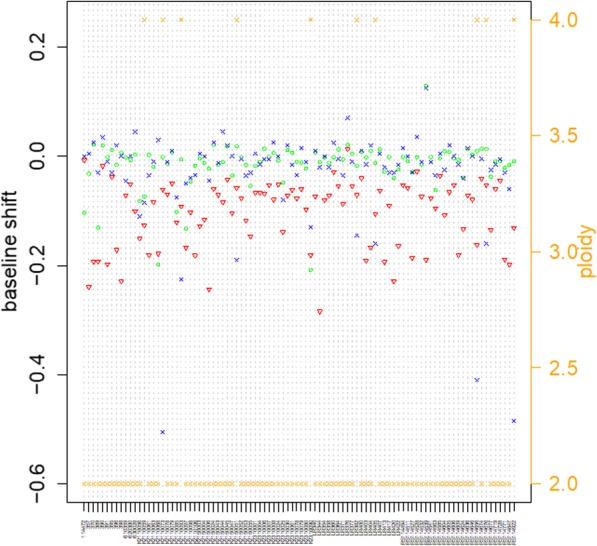
The LRR baseline shift estimation of three SNP based methods and ploidy estimation of GPHMM. Y1 is the baseline shift. Triangle is OncoSNP, circle is GPHMM and blue cross is the GAP. Y2 is the ploidy estimation. Yellow cross in Y2 is the GPHMM

As shown in the Fig. 4, 94 samples’ average LRR baseline shift given by GPHMM, GAP and OncoSNP are − 0.02, − 0.03 and − 0.11. OncoSNP’s prediction shows over aneuploidy. GPHMM and GAP get similar baseline output and their predicting samples’ ploidy level is basically accordance with the pathological report.

**Fig. 5:**
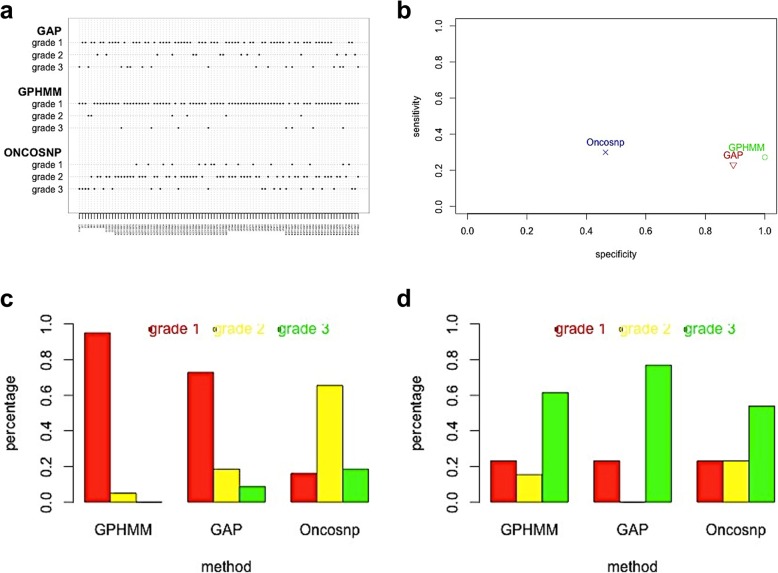
The SNP based methods ranking results. **a** Grades on total 94 samples. **b** Sensitivity and specificity based on the ranking results. **c** Three methods’ comparison on near diploid samples. **d** Three methods’ comparison on over diploid samples

Figure 5a depicts three methods’ ranking results. GPHMM gets most grade 1 on total 94 samples. We further divide the samples into two groups by their ploidy. Shown in the Fig. 5c, for near diploid samples, GPHMM gets quite low grade 3 rate. However, shown in the Fig. 5d, all three methods don’t work well on 14 over diploid samples. The grade 1 rate of three methods doesn’t reach 25%. It indicates that the SNP based methods has no enough power to discriminate the tiny difference of the over diploid samples’ complex BAF and LRR patterns, especially under the existence of noises. OncoSNP is a little more sensitive to aneuploidy than the other two methods, but this sensitivity is at the expense of low specificity, shown in Fig. 5b. About 19% diploid samples are mistakenly predicted as the aneuploidy and at least one chromosome-scale region of over 60% samples is given wrong copy number by OncoSNP. The aberrant baseline shift prediction reflects OncoSNP’s over sensitivity.

#### OncoSNP versus GPHMM

Both based on the HMM model, GPHMM performs much better than OncoSNP. Comparing HMM hidden states in the two methods, OncoSNP has 21 tumor states and is able to detect up to 6n CNAs, while GPHMM has only 12 tumor states and predicts up to 5n CNAs. Although OncoSNP takes more 9 states into consideration, these states mainly distinguish germline LOH with somatic LOH. On the contrary, GPHMM designs one more state for the occasional signal fluctuation, which could tolerate the noise’s influence. The higher true positive rate and specificity on all near diploid samples and being able to process samples of large LRR standard deviation prove that GPHMM applies the HMM model more successfully. OncoSNP produces some obvious wrong results. For example, it predicts a low LRR region with a greater copy number than a high LRR region.

#### GAP versus GPHMM

**Fig. 6:**
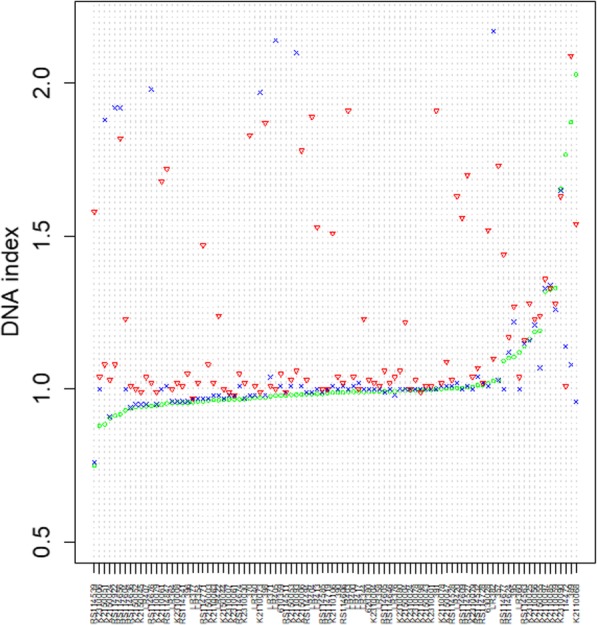
DNA index estimation of three methods on 94 samples. Total 94 samples are sorted in an ascend order according to the GPHMM’s DNA index estimation. Circle symbol represents the GPHMM. Cross symbol is GAP. Triangle symbol is the OncoSNP

GAP gets grade 1 on 75% of near diploid samples. For most of normal cases, GAP and GPHMM perform similarly, which is validated by their similar DNA index output shown in the Fig. 6. The DNA index is originally used by flow cytometer to characterize DNA content of tumor genome relative to normal diploid. In silico, DNA indexes are calculated by averaging segmental copy numbers. GPHMM and GAP predict similar DNA index on 76 of total 94 samples, with the difference less than 0.1.

**Fig. 7:**
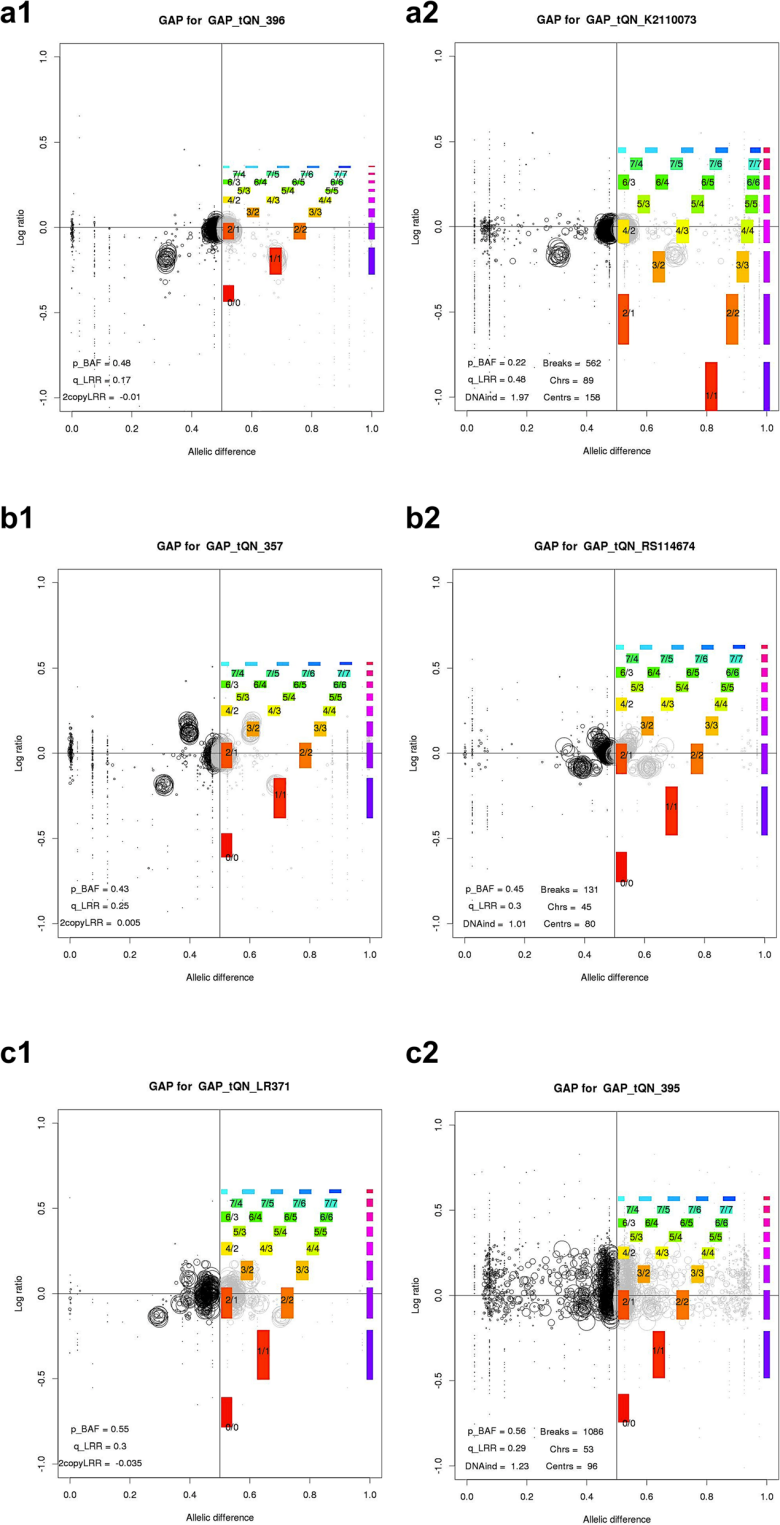
Three cases demonstrate the inherent weaknesses of GAP. **a**1, **a**2 and **b**1, **b**2 are two cases with similiar patterns but different distance between circles. **c**1, **c**2 is the case with unknown typical pattern in GAP

With more comprehensive comparison, the grade 1 rate of GAP is still less than GPHMM by 16%. Mosén thought GAP to be better than GPHMM, but the GPHMM’s author declares that GPHMM has been underestimated. Our results prove that GAP is indeed inferior to GPHMM. In theory, GAP is a kind of pattern recognition method. GAP uses a two-dimension grid as the solution space to cover all possible CNAs configurations. By the side projection of one sample’s BAF and LRR signals, each CNA candidate is represented as a circle in the grid. GAP matches the profile of circles to a template to determine sample’s copy number. GAP’s pattern recognition approach has some inherent weaknesses. Firstly, GAP is not robust and flexible. In the Fig. 7a, samples 396 and K2110073 have quite similar pattern but just a little difference in the BAF and LRR position. It may be caused by different purity. GAP assigns K2110073 as tetraploid, while assigns 396 as diploid. Secondly, GAP is vulnerable to contamination. High contamination makes all circles shrink to the center. In the Fig. 7b, 357 and RS114674 have quite similar pattern. When circles’ boundary distance of sample 357 is large enough, GAP could perfectly identify its CNAs pattern. But for RS114674, when the shrinkage is high and normal state’s circles overlap with those of the copy number gains, GAP fails to get correct result. Thirdly, GAP is incapable of dealing with outliers. In the Fig. 7c, samples LR371 and 395 have large BAF and LRR standard deviation. Such patterns are not the typical known ones for GAP, and thus GAP is unable to recognize them. In contrast, GPHMM could robustly give satisfactory results for the above cases.

Based on above comparison, GPHMM works best among three SNP based methods on near diploid samples. However, no method shows overwhelming advantage over the others on over diploid samples. Ten samples’ corresponding best SNP based CNAs results are chosen as benchmarks for the evaluation of the sequencing based methods.

### Evaluation of sequencing based methods

#### Parameters

The first configuration for most of the sequencing based methods is to cut certain size window for calculation of the read count. Only rSWseq needn’t dividing the genome into small windows in advance. BICseq adopts an aggregation way to segment the genome. Hence it is insensitive to the initial window size setting. CNVseq, FREEC and ReadDepth could automatically discover the optimal window size parameters. FREEC needs specifying the coefficient of variation to determine window size. ReadDepth’s window size is controlled by the false-discovery rate. We use the default setting for all methods except CNVseq. For CNVseq, the *p*-value and the threshold for the gain and loss work together to decide the initial window size. Its default values 10^− 5^ and 0.6 are too stringent for our large coverage data. So we set *p*-value = 0.01 and threshold = 0.2 for CNVseq.

Table 2 lists these 10 samples’ coverage information, the ploidy estimation, the SNP based method’s CNAs result used as benchmark and the estimated windows size of FREEC, CNVseq and ReadDepth. Ten samples’ coverage varies from 47X to 86X. The window sizes of CNVseq and FREEC are inverse proportional to the coverage. But for ReadDepth there is no obvious relationship. Although CNVseq has been accepted as the CNAs caller by many important works [50, 51]. There is a severe error about window size never be reported. In CNVseq source code, the estimated window size would be divided into a half to create a new variable “step”, which is used to coordinate the windows along the genome. When the window size is an odd number, CNVseq will truncate the float number result to an integer number. CNVseq expects the numbers like x.5 always round to the floor, but Perl doesn’t work as this expectation. For example, 0.5, 1.5, 2.5, 3.5, 4.5, 5.5, 6.5, 7.5, 8.5, 9.5 is rounded to 0, 2, 2, 4, 4, 6, 6, 8, 8, 10 in Perl. Therefore, when the step of the window ends with × 1.5, × 3.5, × 5.5, × 7.5 or × 9.5, CNVseq will have no output in the field of “cnv”. Sample K2110056 belongs to this situation. We revise this bug to make CNVseq work on sample K2110056.

**Table 2:** Basic sequencing information and windows size estimation on 10 samples

Sample	Reads		Coverage (fold)		Ploidy	Window size (bp)		
	Tumor	Normal	Tumor	Normal	(ref)	CNVseq	FREEC	ReadDepth
357	1,891,204,418	1,947,625,118	61	63	2 (GPHMM)	1225	618	500
620,380	2,318,387,458	2,660,245,982	75	86	2 (GPHMM)	940	493	600
K2110056	1,665,457,542	1,614,695,950	54	52	2 (GPHMM)	1423	695	600
K2150024	1,468,032,864	1,821,106,022	47	59	2 (GPHMM)	1456	780	600
K2310007	1,707,025,098	1,534,828,476	55	50	2 (GPHMM)	1453	670	600
K2310024	1,795,611,576	1,914,189,016	58	62	2 (GPHMM)	1261	637	600
K2310030	1,565,239,138	1,552,514,106	51	50	2 (GPHMM)	1493	730	600
RS114527	1,750,239,946	1,406,561,492	57	45	4 (OncoSNP)	1517	655	600
K2110097	1,561,020,588	1,821,446,462	50	59	3 (GPHMM)	1725	740	600
K2150051	1,584,801,578	1,523,937,258	51	49	4 (GAP)	1509	726	600

**Fig. 8:**
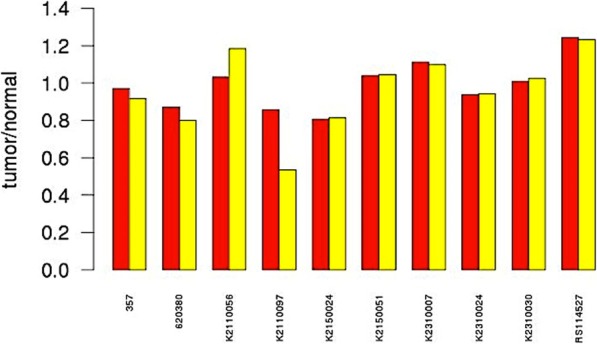
Comparison of the read count ratio between without and with quality filtering

Besides the window size, another parameter is the total read number imbalance adjustment for the matched samples methods. BICseq, FREEC and CNVnorm integrate it as a built-in function. CNVseq needs to swift off the parameter “chromosomal.normalization” for the whole genome scale normalization. rSWseq and Varscan require users to specify the tumor and normal samples’ total read number. When counting the total read number, rSWseq does nothing about the read quality filtering. Varscan only considers the bases meeting phred base quality ≥20. In the Fig. 8, we compare the impact of read’s quality filtering (phred base quality ≥20) on the final tumor/normal total reads ratio. K2110056 and K2110097 are two cases that show different ratios between without and with filtering. Thus, Varscan needs carefully setting the parameter “recenter-up” or “recenter-down” to adjust this imbalance.

#### The recall rate and Jaccard index

For most of samples, GPHMM outputs are used as the benchmark. Only RS114527 and K2150051 take OncoSNP and GAP respectively.

In the Tables 3 and 4, CNVnorm achieves the best recall rate on both near diploid samples and over diploid samples, but it gets the lowest Jaccard Index. CNVnorm predicts 10 samples’ ploidy as 2, 4, 2, 3, 1, 2, 2, 5, 2 and 2. Only 4 samples are accordant with the SNP results. For total 10 samples, the average copy number predicted by CNVnorm is 8. This state overestimation leads to large amount of false positive callings.

**Table 3:** Recall rate of sequencing based methods on 10 samples

Sample	BICseq	CNVnorm	FREEC	CNVseq	rSWseg	Varscan	CNVnator	RDXplorer	ReadDepth
357	0.9	0.97	0.97	0.82	0.45	0.84	0.68	0	0.06
620380	0.97	0.99	0.99	0.92	0.16	0.94	0.15	0.01	0.26
K2110056	0.57	0.98	0.74	0.44	0.23	0.27	0.05	0	0.08
K2150024	0.91	0.93	0.17	0.74	0.02	0.9	0	0.01	0.05
K2310007	0.99	1	0.89	0.96	0	0.98	0.2	0.01	0.01
K2310024	0.99	1	0.88	0.96	0.52	0.97	0.36	0	0.2
K2310030	0.99	0.99	0.94	0.96	0.27	0.98	0.48	0.01	0.7
AVG	0.91	0.98	0.8	0.83	0.24	0.84	0.27	0.01	0.19
RS114527	0.17	0.23	0.74	0.18	0.01	0.17	0.01	0	0.08
K2110097	0.39	0.4	0.26	0.35	0	0.3	0.18	0	0.71
K2150051	0.57	0.94	0.55	0.52	0.01	0.46	0.04	0.01	0.04
AVG	0.38	0.52	0.52	0.35	0.01	0.31	0.08	0	0.28

**Table 4:** Jaccard index of sequencing based methods on 10 samples

Sample	BICSeq	CNVnorm	FREEC	CNVseq	rSWseq	Varscan	CNVnator	RDXplorer	ReadDepth
357	0.78	0.22	0.79	0.59	0.35	0.64	0.57	0	0.05
620380	0.65	0.13	0.66	0.6	0.1	0.73	0.08	0.02	0.04
K2110056	0.45	0.6	0.68	0.32	0.19	0.27	0.07	0	0.08
K2150024	0.73	0.23	0.05	0.55	0.01	0.81	0.01	0.01	0.04
K2310007	0.6	0.05	0.12	0.53	0	0.7	0.19	0.02	0.01
K2310024	0.66	0.06	0.14	0.52	0.41	0.72	0.29	0.01	0.05
K2310030	0.73	0.19	0.49	0.64	0.14	0.85	0.64	0.01	0.43
AVG	0.66	0.21	0.42	0.54	0.17	0.67	0.26	0.01	0.1
RS114527	0.13	0.16	0.69	0.15	0.02	0.14	0.02	0	0.06
K2110097	0.38	0.32	0.26	0.35	0	0.3	0.22	0	0.63
K2150051	0.47	0.28	0.42	0.41	0	0.36	0.03	0.01	0.04
AVG	0.33	0.25	0.46	0.3	0.01	0.27	0.09	0	0.24

**Fig. 9:**
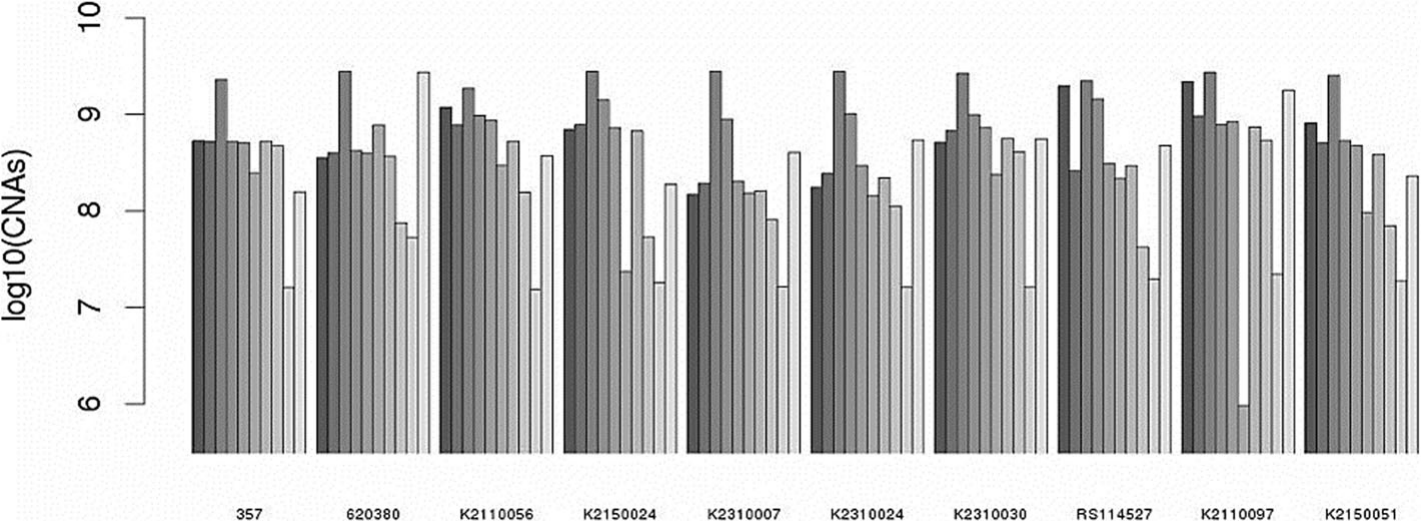
Total CNAs size called by 10 methods on 10 samples. For each group of bars, methods from the left to the right are one SNP based benchmark and 9 sequencing based methods listed in Table 3

**Fig. 10:**
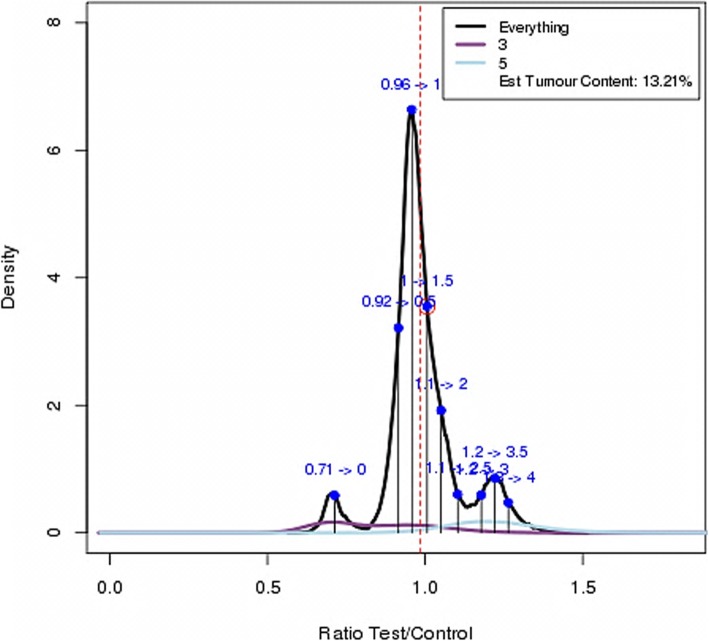
Nine copy number states chosen by the Akaike’s information criterion (AIC) of CNVnorm on sample 357

In the Fig. 9, total output CNAs size from CNVnorm is the largest among all methods. Its low Jaccard index implies that CNVnorm suffers from high false positive rate. We deeply investigate CNVnorm on one sample 357. In fact, 357 only has chromosome 3p loss and chromosome 5 and 7 gain. In Fig. 10, CNVnorm depicts three curves representing the raw read count ratio distributions on the whole genome, chromosome 3 and 5. Its relevant ratio values that assigned to each copy number are labelled on the black color curve. Peaks of the chromosome 3 and 5 are close to the ratios of the estimated copy number 0 and 7(7 = 3.5*2) respectively. The actual copy number gain should start from the estimated copy number 7 in CNVnorm. Therefore, the copy numbers (from 1.5*2 to 3*2) estimated by CNVnorm are false positive predictions.

Putting CNVnorm aside, the other methods perform better on the near diploid samples than on over diploid samples, no matter in terms of the recall or the Jaccard index. On 7 near diploid samples, the means of the best recall rate and the Jaccard index reach 0.94 and 0.75. On 3 over diploid samples, they are 0.67 and 0.6. Like SNP methods, no sequencing based method could work well both on diploid and over diploid samples. BICseq gets the second best recall rate and the Jaccard index on diploid samples, while FREEC performs best on over diploid samples. Varscan gets the best Jaccard index and the third best recall rate on the near diploid samples. Though Varscan is the same with CNVnorm on using the CBS for the segmentation, their performance is dramatically different. Unlike CNVnorm, Varscan doesn’t utilize complex model or processing. Its simple strategy of only focusing on quality control and empirical determining gain and loss cutoff works stably for most normal situations. rSWseg is the only one that needn’t dividing the genome into short windows. Its results are not ideal. In total 6 matched samples methods, rSWseq and CNVnorm are two that perform poorly. They have a similarity of making more assumption or introducing more transformation to explore CNAs. It implies that strong restriction and additional operations may bring a negative effect. If one method couldn’t be self-adaptive to different conditions, it is not only unable to solve the complex cases, but also often decreases the performance in normal cases. About true positive rate, Magi concluded that RDXplorer is better than ReadDepth, CNVseq and CNVnator. Junbo ranked the methods in a descending order as FREEC, RDXplorer, CNVseq and ReadDepth. But our results are quite different from theirs. Especially for RDXplorer, it gets the lowest recall rate. On all 10 samples, RDXplorer calls the fewest variants, which is shown in the Fig. 9. There is one point not mentioned in the works of Magi and Junbo. RDXplorer uses single chromosome rather than the whole genome as a unit to detect an interval’s copy number state. Therefore, RDXplorer is not capable of capture large-scale inter-chromosome events.

At the whole genome level, the single tumor sample based methods couldn’t be comparable with the matched samples based methods. Their average recall rates are much lower than the matched samples based methods. Shown in the Fig. 9, they lose many long and obvious events. If simply ranking the single tumor sample based methods, CNAnator works better on near diploid samples, while ReadDepth is better on over diploid samples.

#### CNAs segmentation

Segmentation is another important issue in CNAs detection. There are two aspects to measure methods’ segmentation performance. One is the breakpoint accuracy and the other is the ability to detect different size of CNAs. Magi and Junbo constructed synthetic dataset to evaluate the breakpoint accuracy. But the synthetic data couldn’t represent the real situation. In fact, segmentation evaluation could only consider the methods’ ability to detect different sizes of CNAs. Because any bias of outputting a certain size of CNAs must lose the breakpoint accuracy. We get insight into 9 sequencing based methods whether are inclined to output special size of CNAs. Each method’s CNAs outputs are divided into five intervals: less than 50Kb, 50-100Kb, 100-500Kb, 500Kb-1 M, and greater than 1 M.

**Fig. 11:**
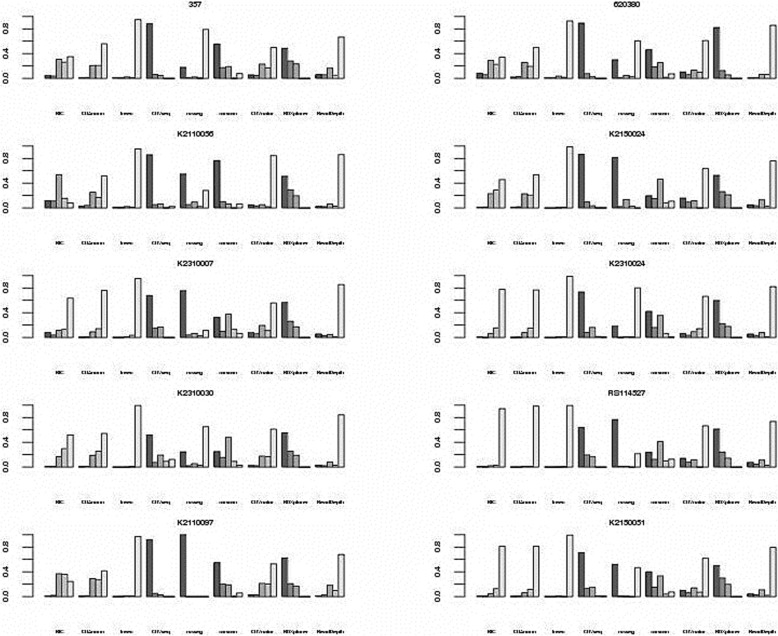
Distribution of CNAs falling in the five size intervals on 10 samples for 9 sequencing based methods’

In the Fig. 11, FREEC, CNVseq and ReadDepth have apparent bias. FREEC and ReadDepth trend to call large size events. For FREEC on 10 samples, the percentages for the over 1 Mb of CNAs taking up the total CNAs size are 0.949, 0.927, 0.954, 0.990, 0.951, 0.988, 0.995, 0.995, 0.974 and 0.994. For ReadDepth, they are 0.666, 0.857, 0.861, 0.759, 0.855, 0.821, 0.843, 0.736, 0.680 and 0.795. On the contrary, CNVseq is inclined to output short CNAs. The percentages for the less than 50Kb of CNAs are 0.887, 0.893, 0.859, 0.864, 0.679, 0.735, 0.518, 0.641, 0.922 and 0.713. The distribution of CNAs size for the other methods is relatively even. ReadDepth’s short events output bias may have a problem of mosaic. When discover the significant recurrent CNAs events in a group of samples, a region consisting of many short events may be lost because these short events just overlap a little. Mosaic effect seldom occurs in the methods biased to large events, because they always merge short events into a long one with lower read count ratio value. But they sacrifice the short events of interest. Varscan is relatively unbiased. BICseq is the only method could manually control segmentation degree. We investigate the relationship between total CNAs size, total CNAs number and size bias through tuning the parameter *λ* of BICseq on sample 357.

**Fig. 12:**
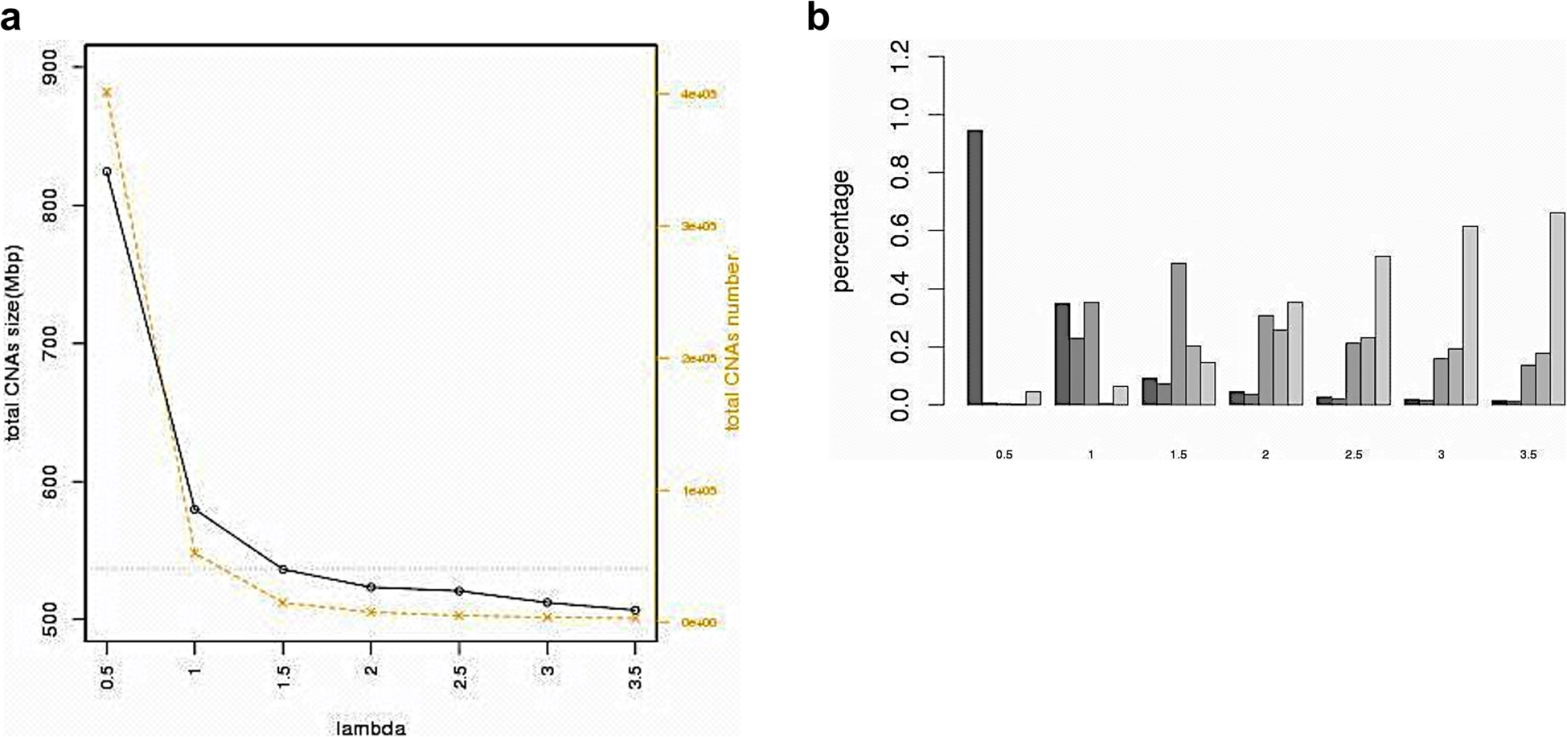
**a** The total size and total number of CNAs change with the *λ* from 0.5 to 3.5. Y1 is the total CNAs size, and Y2 is the total CNAs number. **b** Distribution of CNAs in five size intervals with *λ* from 0.5 to 3.5

In the Fig. 12a, *λ* is inverse proportional to the total CNAs size and CNAs number. At beginning, the small *λ* retains large number of short CNAs. The total CNAs size is 1.5 times of GPHMM result. As *λ* is increasing, some short CNAs are merged by adjacent windows. The total CNAs size and number reduce quickly and the percentages of 50-100Kb and 100-500Kb intervals obviously increase, shown in the Fig. 12b. When *λ* is equal to 1.5, the total size is closest to the reference GPHMM total size. After it, total size and number gradually converge. The CNAs size interval distribution keeps similar shape after *λ* is greater than 2.5.

### Computation efficiency

BAM (Sequence Alignment/Map) is the default file format to store the large nucleotide sequence alignments data. For one 40X human being sample, the BAM size is greater than 100GB. Therefore, the powerful computation and huge storing space are necessary for the sequencing data analysis. Besides the algorithm itself determines the spatial and temporal efficiency, the selected programming language and implementation skills also influence the practical usage.

**Fig. 13:**
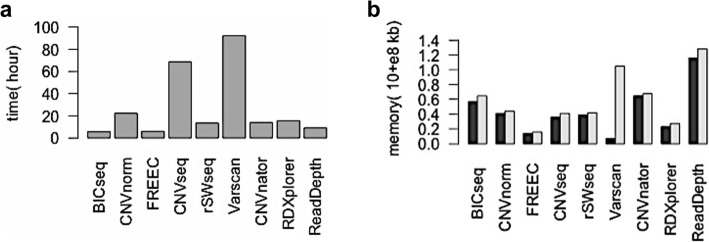
Running time and RAM consumption of nine sequencing based methods. **a** is the running time. **b** is the RAM consumption

We evaluate 9 sequencing based methods’ running time and storing space consummation. Shown in the Fig. 13, among 9 sequencing based methods, FREEC gets the best computation performance, no matter in the aspect of running time or the RAM consumption. C++ shows its advantage in the implementation efficiency. In contrast, another popular programming language Java based tool Varscan needs very long time to calculate the read count ratio. Meanwhile, Java needs more RAM. Strongly recommend to increase the initial and maximum Java heap size before starting Varscan. CNVseq is the second most time-consuming tool. In the R implementation of CNVseq, a loop statement that merges the windows with the same ‘cnv’ number occupies a lot of time. ReadDepth emphasizes that it could carry out in a parallel way by allocating the tasks to multiple cores. It is really quicker than CNAnator and RDXplorer on single sample. But ReadDepth is the heaviest RAM demanding and the only one of 12 methods that isn’t able to process multiple samples simultaneously. ReadDepth fixes the working directory and users have to put the data in the specified locations. Although RXDplorer needs less RAM, it unzips the BAM file and generates a huge temperate SAM file. For a typical BAM file in size 150GB, the corresponding unzipped format file SAM will take up about 1 TB disk space.

## Discussion and conclusion

In this work, we compare total 12 typical CNAs detection methods. Our findings are established on the large-scale real dataset at the whole genome level. This is the most difference between our comparison and other comparison works. According to our comparison findings, there’s no method could work well in all scenarios. We recommend the following principles to choose the optimal method for the given situation. For SNP array based methods, we could trust GPHMM for most near diploid samples. OncoSNP could accompany with GPHMM and works for over diploid samples. GAP is suitable for regular cases and provides a good visualization output to facilitate the manual inspection. All three methods couldn’t directly identify somatic CNAs. GAP carefully annotates the SNP probes and the centromeres position before calling CNAs. GAP won’t mistakenly call centromeres as CNAs. But this situation exists in the GPHMM and OncoSNP. GPHMM and OncoSNP need post-processions to filter out these callings. With regards to the sequencing based methods, CNVnorm and rSWseq aren’t recommended for their inherent weaknesses. BICseq is suitable for diploid samples analysis and Varscan is an alternative option for its good specificity and even segmentation. FREEC is recommended for over diploid samples. Although FREEC has strong bias to large size output, large events account for the most parts of over diploid samples’ CNAs. If the sequencing experiments have enough high quality and investigators expect to discover short CNAs events, CNAseq is qualified for this purpose. One point must be emphasized that the single tumor sample based methods with GC-content correction couldn’t rival the matched samples based methods on the somatic CNAs detection. The single tumor sample based methods are only applicable under specific conditions such as no matched normal sample. CNAnator is suitable for near diploid samples and ReadDepth is for over diploid samples. RDXplorer is only able to detect the events on single chromosome.

There are still a lot of aspects need the upcoming sequencing based methods to improve. First, it should be self-adaptive to deal with near diploid and over diploid samples. Normally, investigators couldn’t know whether their samples suffer from aneuploidy in advance. So methods should automatically estimate and adjust their parameters for different ploidy conditions. Second, it should be capable of estimating the exact copy number, which could help intratumoral heterogeneity recognition. Although CNVnorm took a try, it wasn’t successful. Third, it should improve the contamination estimation. One specimen needs processing by the library preparation, PCR duplication and so on. They all input noise and increase the difficulties of contamination detection.

## Data Availability

Not applicable.

## Abbreviations

aCGH: array comparative genomic hybridization
BAF: B Allele Frequency
CAGEKID: CAncer GEnomic of the KIDney
CNAs: Copy number alterations
CNVs: Copy number variations
ICGC: International cancer genome consortium
LOH: Loss of Heterozygosity
LRR: Log R Ratio
SNP: Single nucleotide polymorphism
TCGA: The cancer genome atlas
